# Preclinical Comparison of Stem Cells Secretome and Levodopa Application in a 6-Hydroxydopamine Rat Model of Parkinson’s Disease

**DOI:** 10.3390/cells9020315

**Published:** 2020-01-28

**Authors:** Fábio G. Teixeira, Helena Vilaça-Faria, Ana V. Domingues, Jonas Campos, António J. Salgado

**Affiliations:** 1 Life and Health Sciences Research Institute (ICVS), School of Medicine, University of Minho, 4710-057 Braga, Portugal; helena.mv.faria@gmail.com (H.V.-F.); campos.jonas@hotmail.com (J.C.); 2 ICVS/3B’s Associate Lab, PT Government Associated Lab, 4806-909 Braga/Guimarães, Portugal

**Keywords:** Parkinson’s Disease, mesenchymal stem cells, stem cells secretome, levodopa

## Abstract

Parkinson’s Disease (PD) is characterized by the massive loss of dopaminergic neurons, leading to the appearance of several motor impairments. Current pharmacological treatments, such as the use of levodopa, are yet unable to cure the disease. Therefore, there is a need for novel strategies, particularly those that can combine in an integrated manner neuroprotection and neuroregeneration properties. In vitro and in vivo models have recently revealed that the secretome of mesenchymal stem cells (MSCs) holds a promising potential for treating PD, given its effects on neural survival, proliferation, differentiation. In the present study, we aimed to access the impact of human bone marrow MSCs (hBM-MSCs) secretome in 6-hydroxydopamine (6-OHDA) PD model when compared to levodopa administration, by addressing animals’ motor performance, and substantia nigra (SN), and striatum (STR) histological parameters by tyrosine hydroxylase (TH) expression. Results revealed that hBM-MSCs secretome per se appears to be a modulator of the dopaminergic system, enhancing TH-positive cells expression (e.g., dopaminergic neurons) and terminals both in the SN and STR when compared to the untreated group 6-OHDA. Such finding was positively correlated with a significant amelioration of the motor outcomes of 6-OHDA PD animals (assessed by the staircase test). Thus, the present findings support hBM-MSCs secretome administration as a potential therapeutic tool in treating PD, and although we suggest candidate molecules (Trx1, SEMA7A, UCHL1, PEDF, BDNF, Clusterin, SDF-1, CypA, CypB, Cys C, VEGF, DJ-1, Gal-1, GDNF, CDH2, IL-6, HSP27, PRDX1, UBE3A, MMP-2, and GDN) and possible mechanisms of hBM-MSCs secretome-mediated effects, further detailed studies are needed to carefully and clearly define which players may be responsible for its therapeutic actions. By doing so, it will be reasonable to presume that potential treatments that can, per se, or in combination modulate or slow PD may lead to a rational design of new therapeutic or adjuvant strategies for its functional modeling and repair.

## 1. Introduction

Parkinson’s Disease (PD) represents the second most prevalent (worldwide) neurodegenerative disorder and is characterized by the progressive degeneration of dopaminergic neurons (DAn) in several dopaminergic networks [1]. As a consequence, patients develop several motor complications including rigidity, bradykinesia, and tremors [2]. Mitochondrial dysfunction, excessive reactive oxygen species (ROS), and ubiquitin-proteasome (UPS) impairment have been consistently described as involved in the mechanisms responsible for DAn degeneration [3]. The loss of dopamine (DA), irrespectively of the causal chain of events, underlies the reasoning for the gold standard treatment of PD, which still is the administration of levodopa [4]. Nevertheless, although the application of such treatment is efficient in reducing the resting-tremors and other primary motor symptoms during the first years of the disease, it remains, however, insufficient to preserve, or replace degenerated DAn or to stop further progression of PD [5,6,7]. Furthermore, its extended use, frequently associated with an increase in dosage due to the natural progression of the disease, has been associated with the appearance of nausea, vomiting, low blood pressure, restlessness, drowsiness or sudden onset of sleep, as well as impulsive and addiction behavioral changes [5]. This ‘pathophysiological’ effect of levodopa has been described as being due to a combination of disease-related factors, as well as to levodopa pharmacokinetics itself [8]. Actually, while the correlation to disease factors remains unclear, the unusual pharmacokinetic characteristics of levodopa, per se, have been correlated with motor complications pathogenesis (e.g., dyskinesia’s). Indeed its (very) short half-life leads to abnormal pulsatile stimulation of DA receptors, inhibiting the establishment of a correct and effective balance of the nigrostriatal pathway [8]. Therefore, there is an urgent need to find novel therapeutic strategies that can overcome the limitations posed by levodopa, not only to delay the progression of PD but also to improve and maintain PD patients’ quality of life. 

One such alternatives is cell transplantation strategies that profit from the potential regenerative properties provided by the use of stem cells. In accordance, mesenchymal stem cells (MSCs) have themselves been proposed as a promising therapeutic tool for PD [9] given their ability to modulate DAn survival and differentiation [1]. Actually, MSCs have been defined as a multipotent stem cell population with a great therapeutic potential for several diseases, being available widespread throughout the human body by presenting a great proliferative potential with minimal senescence through multiple passages [1]. According to the International Society for Cellular Therapy (ISCT) there are minimal criteria to identify MSC populations such as (1) the adherence to plastic in standard culture conditions; (2) the positive expression of specific markers like CD73, CD90, CD105, and negative expression of hematopoietic markers like CD34, CD45, HLA-DR, CD14, or CD11B, CD79α or CD19, as well as (3) the in vitro differentiation into at least adipocytes, osteoblasts, and chondroblasts [10]. Notwithstanding, recent evidence has claimed that these criteria do not totally support a complete purification of homogenous MSC populations, assuming that the isolation of MSCs according to the ISCT criteria produces heterogeneous, nonclonal cultures of stromal cells containing stem cells with different multipotential properties, committed progenitors, and differentiated cells [11]. Therefore, although the nature and function of pure MSCs remain to be explored, the therapeutic effects of them under the current definition have demonstrated promising outcomes both from pre-clinical and clinical points of view [12].

These beneficial effects have been attributed to their secretome, which has been described as a complex mixture of soluble products composed by a proteic soluble fraction (constituted by growth factors and cytokines), and a vesicular fraction composed by microvesicles and exosomes, which are involved in the transference of proteins and genetic material (e.g., miRNAs) to other cells, with promising therapeutic effects [12]. In addition to this, recent studies have indicated that rather than being a regular mixture of molecular bioactive molecules, MSCs paracrine activity is known to be dependent on the diverse stimuli present in the microenvironment in which MSCs are located or delivered [13]. Being so, studies have indicated that the composition of MSCs secretome can be modulated by preconditioning them during in vitro culture [13].

Our lab has shown that the sole injection of MSCs secretome discloses the same effects to those observed in MSC cell-transplanted groups in the improvement of neuronal and glial densities, thereby indicating that MSCs secretome per se can be used as a ready-to-use product for therapeutic purposes [14]. Furthermore, we have succeeded in modulating MSCs secretome using dynamic culture conditions through computer-controlled bioreactors [15], which when locally applied in the substantia nigra (SN) and striatum (STR) of a 6-hydroxydopamine (6-OHDA) unilateral PD rat model, had a significant effect on the survival of DAn, a fact that was positively correlated with animal motor performance amelioration [16]. This is, therefore, the right timing to address MSCs secretome as a promising therapeutic tool in treating PD, by comparing it to levodopa. Thus, in the present work, we addressed the impact of MSCs secretome on DAn cell survival and motor function of a 6-OHDA animal model of PD by comparing it to levodopa administration, exploring possible mechanisms and pathways having in mind the neuroregulatory profile of MSCs secretome.

## 2. Methods

### 2.1. Expansion of Human Bone Marrow MSCs (hBM-MSCs) and Secretome Collection

Pre-isolated and cryopreserved hBM-MSCs were thawed at 37 °C and plated in a T-75-treated polystyrene flask culture with 15 mL of α-MEM growth medium (containing 1% of antibiotic/antimycotic and 10% of fetal bovine serum (FBS) (Invitrogen, Waltham, MA, USA). The cell culture medium was renewed every 3 days and the culture maintained at 37 °C, 5% CO_2_, 95% air, and 90% relative humidity until confluence. After 80–90% of cell confluence, the cells were enzymatically digested with 0.05% trypsin for 5 min at 37 °C. α-MEM growth medium was used to stop the reaction. Following that, the cells were centrifuged at 1200 rpm for 5 min, the pellet was resuspended, and 5000 cells/cm^2^ were plated into new cell culture flasks and the procedure was repeated until cells reached passage 5 (P5). Therefore, at P5 and for secretome collection (e.g., in the form of conditioned medium (CM), 5000 cells/cm^2^ were plated and kept for 3 days in α-MEM medium (Invitrogen, USA) supplemented with 1% of antibiotic/antimycotic (Invitrogen, USA) and 10% FBS (Invitrogen, USA). After this, the flasks were washed three times in Neurobasal A medium (Invitrogen, USA) for 5 min and then, washed five times in PBS without Mg^2+^/Ca^2+^ (Invitrogen, USA). Neurobasal A medium supplemented with kanamycin (1%) (Invitrogen, USA) was added and 24 h after, the medium was collected and frozen at −80 °C until use.

### 2.2. 6-Hydroxydopamine (6-OHDA)-Induced Lesions and hBM-MSCs Secretome and Levodopa Administration

**Figure 1:**
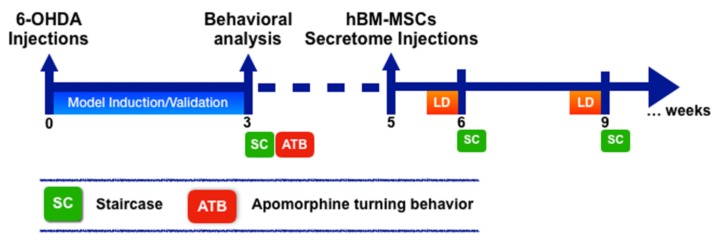
Experimental design. A unilateral 6-hydroxydopamine (6-OHDA) injection into the medial forebrain bundle (MFB) was performed to induce the rat Parkinson’s Disease (PD) model. Animal behavioral analysis through rotameter and staircase behavioral tests was performed to validate the model 3 weeks after 6-OHDA injections. Afterwards, human bone marrow mesenchymal stem cells (hBM-MSCs) secretome was (5 weeks after 6-OHDA injections) locally administrated into the substantia nigra (SN) and striatum (STR), respectively. Levodopa (LD), in turn, was given by oral gavage. 1 and 4 weeks after this treatment procedures, fine motor behavioral assessment (i.e., staircase) was performed.

All the surgical procedures, from the establishment of the 6-OHDA PD model to the surgical administration of hBM-MSCs secretome, were performed as we previously described [16]. Briefly, nine-week-old Wistar Han male rats (≈300 g; Charles River, Barcelona, Spain) were housed (two per cage) and maintained in a controlled environment at 22–24 °C and 55% humidity, on 12 h light/dark cycles and fed with regular rodents’ chow and tap water ad libitum. A week prior to the beginning of injections animals handling was performed, aiming to reduce the stress induced by the surgical procedures. All these manipulations were done in accordance with the Portuguese National Authority for animal experimentation, Direção Geral de Veterinária (ID: DGV28421) and the regulations on animal care and experimentation (European Union Directive 2010/63/EU). Therefore, for 6-OHDA-induced lesion, ketamine-medetomidine (75 mg/kg; 0.5 mg/kg, intraperitoneally (IP)) anesthesia was used, animals (*n* = 35) were placed on a stereotaxic frame (Stoelting, Wood Dale, IL USA), and unilaterally injected, using a 30-gauge needle Hamilton syringe (Hamilton, Bonaduz, CH, Switzerland), with either vehicle (Sham group, *n* = 9) or 6-OHDA (Sigma, *n* = 26) directly into the medial forebrain bundle (MFB) (coordinates related to Bregma: AP = −4.4 mm; ML = 1.0 mm; DV = −7.8 mm [17]; according to Paxinos and Watson brain atlas [18]). Therefore, sham animals received 2 µL of 0.2 mg/mL of ascorbic acid in 0.9% of NaCl and, 6-OHDA animals were injected with 2 µL of 6-OHDA hydrochloride (4 µg/µL) with 0.2 mg/mL of ascorbic acid in 0.9% of NaCl at a rate of 1.0 µL/min. After each injection, the needle was left in place for 4 min in order to avoid any backflow up the needle tract. Three weeks after this procedure, a behavioral assessment using the staircase and apomorphine turning behavior was performed to validate the model (Figure 1).

Concerning treatment procedures, 5 weeks after 6-OHDA PD model induction animals received hBM-MSCs secretome and levodopa. Therefore, 6-OHDA animals were intracranially injected with the vehicle, namely Neurobasal^®^-A medium with kanamycin (6-OHDA group; *n* = 10), and with hBM-MSC secretome (Secretome group; *n* = 8) directly in the SNc and STR as we have previously described [16]. Regarding levodopa treatment (*n* = 8), Sinemet^®^ tablets (Sinemet^®^, 100/25 levodopa/carbidopa, Merck, Sharp and Dohme, S.p.A, Italy) were crushed in water and given by oral gavage, 12 mg/kg and 1 h before behavioral assessment as we previously performed [19]. At 1 and 4 weeks, behavioral assessment was performed (Figure 1).

### 2.3. Staircase Test

To access animals’ skilled forelimb motor function, the staircase behavioral test was performed as we have previously described [20]. Briefly, five pellets were placed into each step/well of the double staircase apparatus. In the first 2 days, the animals were subject to a training session, familiarizing with the test apparatus and pellets, which were available for 5 and 10 min, on days 1 and 2, respectively. After that, and during (test session) five consecutive days, animals were kept inside the box and bilaterally exposed to food pellets, having 15 min to reach, retrieve, and eat those pellets present on the steps. Finally, in the last 2 days of testing, animals were exposed to a forced-choice task (FC), having pellets of food-restricted just to one of the steps’-sides (e.g., right (FCR) and left (FCL)). All the sessions were performed at the same time of day and with food-restricted animals. After each test interval, animals were removed from staircase boxes and the remaining (leftover) pellets were counted.

### 2.4. Apomorphine Turning Behavior

To access dopaminergic nigrostriatal integrity after 6-OHDA-induced lesions (and validate the model), apomorphine-induced turning behavior (also known as rotameter behavioral test) was performed. Animals’ necks were subcutaneously injected with a dopamine agonist namely through a 0.05 mg/kg apomorphine hydrochloride (Sigma, St. Louis, MO, USA) solution dissolved in 1% of ascorbic acid in 0.9% of NaCl as we have previously demonstrated. After this, the number of contralateral rotations was digitally recorded, allowing the assessment of vehicle and 6-OHDA injections. Apomorphine was just used to this purpose, and its repeated use was not provided, as it could lead to an overstimulation of the dopaminergic system, and lead to an inadequate interpretation of hBM-MSCs secretome and levodopa effects on the functional outcomes of 6-OHDA PD animals [21,22,23].

### 2.5. Tyrosine Hydroxylase (TH) Immunostaining and Striatal Fiber Density Measurement

Ten weeks after the development of the 6-OHDA PD model and consequent treatment application and behavioral analysis, animals were euthanized with a sodium pentobarbital (Eutasil, 60 mg/kg i.p. Ceva Saúde Animal, Portugal) injection, and transcardially perfused with 4% paraformaldehyde (Merck, Lisbon, Portugal) in 0.1 M PBS. For histological procedures and analysis, striatal and mesencephalon coronal sections (containing SN) with 30 µm of thickness were obtained with a vibratome (VT1000S, Leica, Germany), TH staining being performed as we previously described [16]. Briefly, and for TH-positive cell quantification on the SN, six identical TH-labeled slices spanning the entire mesencephalon were chosen, including all the portions of the SN. Using a bright-field microscope (BX51, Olympus, Center Valley, PA, USA) equipped with a digital camera (PixeLINK PL-A622, CANIMPEX Enterprises Ltd., Halifax, NS, Canada), and with the help of Visiomorph^TM^ software (V2.12.3.0, Visiopharm, Hørsholm, Denmark), the boundaries of the SN area were drawn. The delineation of this region was performed through the identification of anatomic standard reference points with the help of rat brain atlas [18], and the counting of total TH-positive cells in the SN area was performed on both hemispheres. Data is presented as the percentage (%) of remaining TH-positive cells in the injected side, compared to the control side.

Concerning TH immunoreactive striatal fibers, the total immunoreactivity of all TH fibers was measured by densitometry as described by Febbraro et al. [24]. Therefore, four TH-immunostained prosencephalon sections representing the coordinates of injection sites within the striatum were selected and photographed as we previously demonstrated [16]. Photos were converted to greyscale using Image J program (1.51 version; National Institute of Health, Bethesda, MD, USA) and analyzed for grey intensity after calibrating the Image J program, by accessing the optical density (O.D.) of the selected sections and by following the program instructions. Shortly, striatum O.D. values were determined in both brain hemispheres using a 1.1 mm^2^ rectangular grid, including the injection coordinates, as determined by anatomical references and rat brain atlas [18]. Corpus callosum O.D. measurement was also performed in both brain hemispheres, using it as an internal control to avoid nonspecific background. Therefore, TH-positive striatal fiber densities were determined by calculating the O.D. difference between the striatum treated with hBM-MSCs secretome, levodopa, and Neurobasal^®^-A medium with the intact striatum, in which the extension of the immunostaining on the lesioned side was expressed as a percentage of the intact side.

### 2.6. Statistical Analysis

Statistical evaluation for animal apomorphine behavioral test (after 6-OHDA) injection was performed by Student’s *t*-test. For the staircase test, upon 6-OHDA and even hBM-MSCs secretome, levodopa or Neurobasal^®^-A medium injections, ANOVA repeated measures followed by post-hoc Bonferroni for multiple comparisons was performed using SPSS statistic program (version 25; IBM Co., Armonk, NY, USA). Graphical representation by using GraphPad Prism ver.8 (GraphPad Software, La Jolla, CA, USA). Equality of variances and sphericity were measured using the Levene’s and Mauchly’s tests, respectively, and was assumed when *p* > 0.05. Data are presented as mean ± SEM. The significance value was set at *p* < 0.05. Effect size was calculated using η^2^ partial.

## 3. Results

### 3.1. 6-OHDA PD Model Phenotypic Validation

**Figure 2:**
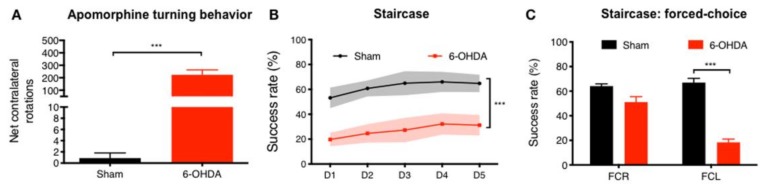
6-OHDA PD model phenotypic validation. Using the apomorphine turning behavioral test, it was verified that after 3 weeks of 6-OHDA injections, there was (**A**) an intense turning behavior promoted by the injection of apomorphine when compared to Sham group, demonstrating that animals’ dopaminergic integrity was affected. Additionally, through the staircase test, it was verified that after 6-OHDA administration there was a (**B**,**C**) significant reduction on fine motor coordination of the tested animals. For apomorphine turning behavioral test Sham *n* = 9, 6-OHDA *n* = 18. For staircase Sham *n* = 9, 6-OHDA *n* = 18. Data presented as mean ± S.E.M. *** *p* < 0.001. FCR—forced choice task at right side; FCL—forced choice task at left side.

To access nigrostriatal functional integrity after 6-OHDA administration, we used the apomorphine turning behavioral test. From the results, and 3 weeks after, we observed that there was a significantly higher number of net-contralateral rotations in the 6-OHDA-injected animals when compared to the Sham group (*t* = 5.561; *p* < 0.001; Figure 2A). Regarding motor performance, which was addressed by the staircase test, we also observed motor deficits after 6-OHDA injections. Of note, when we assessed the forelimb use and skilled motor function, 6-OHDA-injected animals were clearly affected when compared to the control group (Sham; F_(1,25)_ = 222,82; *p* < 0.0001; η^2^_partial_ = 0.899, Figure 2B). Finally, by performing a forced-choice task (in which animals are forced to choose one of the steps-side), we found a significant impairment on the affected side (left-side) when compared to the control group (Sham; right side (FCR): *t* = 2.02, *p* = 0.0536; and left side (FCL) *t* = 10.93; *p* < 0.001, Figure 2C).

### 3.2. hBM-MSCs Secretome Improves 6-OHDA PD Motor and Histological Deficits 

**Figure 3:**
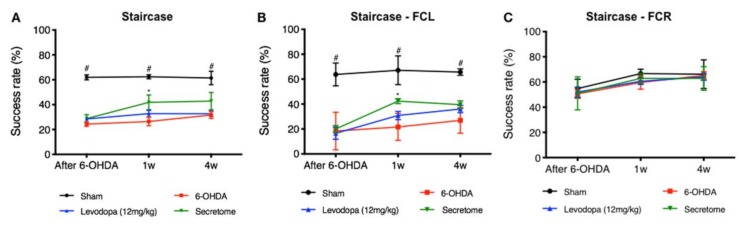
Impact of hBM-MSCs secretome and levodopa interventions on motor coordination performance of 6-OHDA PD animals—staircase. (**A**) The paw reaching motor coordination performance of the animals demonstrated a significant amelioration (at one week, *p* < 0.05) of the forelimb coordination of the hBM-MSCs secretome-injected animals when compared to the untreated group 6-OHDA. Even under a (**B**,**C**) paw reaching forced-task, the animals injected with hBM-MSCs secretome presented a better performance (at one week after injection; *p* < 0.05) when compared to the untreated group 6-OHDA on the (**B**) (left) affected side (FCL). Such pieces of evidence, were, in turn, not observed in the levodopa-treated animals when compared to the untreated group 6-OHDA (**A**–**C**). Sham *n* = 9, 6-OHDA *n* = 7, Levodopa *n* = 5, and hBM-MSCs secretome *n* = 6. Data presented as mean ± S.E.M. *hBM-MSCs secretome-injected animals statistically different from 6-OHDA, *p* < 0.05; ^#^Sham animals statistically different from 6-OHDA, hBM-MSCs secretoma and levodopa-injected animals, *** *p* < 0.001. FCR—forced choice task at right side; FCL—forced choice task at left side.

To access the therapeutic effects of hBM-MSCs secretome and levodopa in 6-OHDA PD animals, animal motor performance was assessed at 1 and 4 weeks through the staircase test. From the results, statistical analysis revealed that after hBM-MSCs secretome administration, there was a significant between-subjects effect (i.e., factor treatment: F_(3,23)_ = 37.58; *p* < 0.001; η^2^_partial_ = 0.831), also for the within-subjects effect (i.e., factor time: F_(2,46)_ = 3.99; *p* = 0.025; η^2^_partial_ = 0.148), but no interaction between these factors (F_(6,46)_ = 1.27; *p* = 0.290; η^2^_partial_ = 0.142). Comparing the hBM-MSCs secretome injected animals with the untreated group (6-OHDA), post-hoc analysis revealed that the administration of the secretome led to a significant amelioration of animal’s motor performance after 1 week (*p* = 0.036, Figure 3A). Additionally, and concerning the forced-choice task procedure, animals injected with hBM-MSCs secretome also had a significant motor amelioration. Statistical analysis revealed an effect for the factor treatment (F_(3,20)_ = 45.84; *p* < 0.001; η^2^_partial_ = 0.873), also for the factor time (F_(2,40)_ = 18.58; *p* < 0.01; η^2^_partial_ = 0.482) as well as interaction between these factors (F_(6,40)_ = 3.11; *p* = 0.013; η^2^_partial_ = 0.318). Considering hBM-MSCs secretome effects, post-hoc testing revealed an increased success rate of eaten pellets in the affected side comparing to the untreated group (6-OHDA, left side, *p* = 0.046, Figure 3B; right side, *p* = 0.945, Figure 3C). Concerning levodopa, post-hoc analysis revealed no differences when compared to the untreated group (6-OHDA; *p* = 0.742; Figure 3A), as well as when compared to the hBM-MSCs secretome (*p* = 0.457; Figure 3A). The same observation was also seen in the forced choice task both for the untreated group (6-OHDA; left side, *p* = 1.0 right side, *p* = 1.0; Figure 3B,C) and hBM-MSCs secretome (left side, *p* = 0.995; right side, *p* = 1.0; Figure 3B,C).

**Figure 4:**
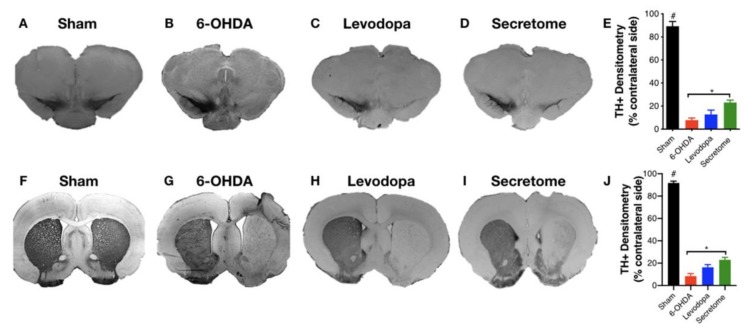
Striatum (STR) and Substantia nigra (SN) brain slices photomicrographs stained for Tyrosine Hydroxylase (TH). Compared to the (**A**,**F**) Sham group, all the animals injected with 6-OHDA had a significant reduction on the TH-positive staining both in the SN and STR. Notwithstanding, animals treated with the (**D**,**I**) hBM-MSC secretome display a significant impact on STR and SN histological deficit, with more TH-positive cells and fibers being observed when compared to (**B**,**G**) untreated group 6-OHDA (**E**,**J**). Concerning levodopa (**C**,**H**), although a slight increase was observed, no differences were observed when compared to the untreated group 6-OHDA (**B**,**G**) and hBM-MSCs secretome (**D**,**I**) both in the SN and STR (**E**,**J**), respectively. Data presented as mean ± S.E.M. Sham *n* = 9, 6-OHDA *n* = 7, Levodopa *n*= 5, and hBM-MSCs secretome *n* = 6; * hBM-MSCs secretome-injected animals statistically different from 6-OHDA, *p* < 0.05. ^#^Sham animals statistically different from 6-OHDA, levodopa, and hBM-MSCs secretome-treated animals, *p* < 0.001. Scale-bar: for SN 200 µm; for STR: 1 mm.

To further analyze the impact of 6-OHDA-induced DAn degeneration, as well as the resulting hBM-MSCs secretome and levodopa interventions, we proceeded to histological analyses for TH staining. From the results, we found that after 6-OHDA injections, there was a significant loss of DAn into the SN (Figure 4B–D) when compared to the control group (Sham, Figure 4A). Notwithstanding, after therapeutic intervention, statistical analysis revealed an effect for the factor treatment (F_(3,18)_ = 153.70; *p* < 0.001; η^2^_partial_ = 0.962) in which the local administration of hBM-MSCs secretome potentiated the survival of DAn, as a significant number of TH-positive cells was observed in the SN when compared to the untreated group (*p* = 0.019, Figure 4E). In contrast, concerning levodopa administration, no effects were observed when compared to the untreated group (*p* = 0.693), and to the treated with hBM-MSCs secretome (*p* = 0.242; Figure 4E). The same trend was also observed for the TH-positive terminals in the striatum (Figure 4G–I), which was assessed by densitometry analysis. Post-hoc analysis revealed an effect for the factor treatment (F_(3,18)_ = 348.76; *p* < 0.001; η^2^_partial_ = 0.983; Figure 4J), demonstrating that the administration of the hBM-MSCs secretome directly in the STR was able to enhance TH-positive terminals when compared to the untreated group 6-OHDA (*p* = 0.01, Figure 4J). Nevertheless, such significance was not observed when compared to levodopa (*p* = 0.304; Figure 4J). Concerning levodopa treated animals per se, as it was observed for the SN, no significant effects were also observed for the TH-positive terminals in the striatum when compared to those untreated (*p* = 0.090; Figure 4J).

## 4. Discussion

Currently, it is widely accepted that the ability to modulate, slow, or preferably stop PD progression is a major scientific and clinical need [25]. Such a gap could be explained by the multifactorial profile of the disease (e.g., alpha-synuclein accumulation, mitochondrial and UPS dysfunction, oxidative stress, and neuroinflammation), which leads to the extensive loss of DAn in the nigrostriatal pathway, thereby resulting in debilitating motor limitations [2] In the in vivo experiments performed in the present study, we used a classical PD rat model, induced by unilateral injection of 6-OHDA into the MFB [17,20]. As previously shown by our group in similar experiments [16,26], the model was successfully validated, by presenting impairments in the dopaminergic nigrostriatal integrity as shown in the rotameter behavioral test (Figure 2A), with the animals displaying an intense turning behavior when compared to the control group (Sham; injected with vehicle). Still, and by addressing fine motor coordination of the 6-OHDA PD animals, such capability was also found to be significantly diminished, as the animals presented deficits in skilled motor functions as evidenced by the staircase test, respectively (Figure 2B,C).

Considering the effects of the treatment pipeline used, it should be highlighted that on the skilled motor function (staircase), hBM-MSCs secretome was the only group found to increase the animals’ success rate of eaten pellets, when compared to control (untreated) animals (6-OHDA; Figure 2A). Additionally, in the forced-choice task and after one week as well, hBM-MSC secretome has potentiated (in the affected side) the paw reaching motor performance of 6-OHDA PD animals when compared to the untreated group (Figure 2B). Moreover, we have also observed that hBM-MSCs secretome administration increased TH-positive neurons and fibers both in the SN and STR (Figure 4), thereby reinforcing the positive skilled motor function amelioration observed (Figure 3). These outcomes nicely correlate with our previous results, demonstrating that stem cells secretome may start being considered a potentially active component in PD modeling and repair [16,26].

**Figure 5:**
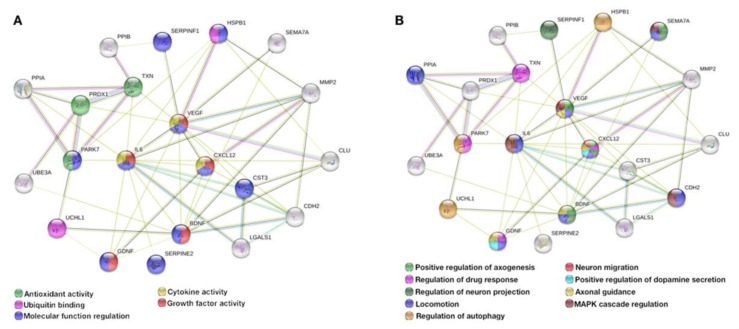
hBM-MSCs are producers of neuroregulatory molecules impacting PD. From previously generated proteomic databases derived from Bioplex-Luminex and Mass Spectrometry-based analysis, we were able to identify the presence of several (neuro)trophic factors and cytokines with important roles and therapeutic actions on PD. Of note, by STRING (Search Tool for the Retrieval of Interacting Genes/Proteins) bioinformatics research tool analysis, from those databases we identified a cluster of 21 interconnected proteins, namely Trx1 (TXN), SEMA7A, UCHL1, PEDF (SERPINF1), BDNF, Clusterin (CLU), SDF-1 (CXCL12), CypA (PPIA), CypB (PPIB), Cys C (CST3), VEGF, DJ-1 (PARK7), Gal-1 (LGALS1), GDNF, CDH2, IL-6, HSP27 (HSPB1), PRDX1, UBE3A, MMP-2, and GDN (SERPINE2), which from the (**A**) biological processes and (**B**) molecular function analysis revealed important actions to PD modeling and repair.

Taking advantage of the proteomic databases previously generated by our group [15,16,27], and in order to further explore possible underlying mechanisms behind the secretome effects, by using the STRING (Search Tool for the Retrieval of Interacting Genes/Proteins) bioinformatics tool, we found a (PD) protein association network composed by 21 proteins, namely Thioredoxin-1 (Trx1/TXN), Semaphorin-7A (SEMA7A), Ubiquitin carboxy-terminal hydrolase L1 (UCHL1), Pigment epithelium-derived factor (PEDF/SERPINF1), Brain-derived neurotrophic factor (BDNF), Clusterin (CLU), Stromal cell-derived factor 1 (SDF-1/CXCL12), Cyclophilin A (CypA/PPIA), Cyclophilin B (CypB/PPIB), Cystatin C (Cys C/CST3), Vascular endothelial growth factor (VEGF), Protein Deglycase DJ-1 (PARK7), Galectin 1 (Gal-1/LGALS1), Glial cell line-derived neurotrophic factor (GDNF), Cadherin 2 (CDH2), Interleukin 6 (IL-6), Heat shock protein 27 (HSP27/HSPB1), Peroxiredoxin 1 (PRDX1), Ubiquitin-protein ligase 3A (UBE3A), Metalloproteinase 2 (MMP-2), and glial-derived nexin (GDN/SERPINE2)—Figure 5. From this association network, we found a cluster of 13 proteins directly interconnected, which from the biological processes (Figure 5A) and molecular function (Figure 5B) were found to have important actions having in mind the dopaminergic system and PD.

Accordingly, and regarding Trx1, it plays a vital role in the maintenance of a reduced intracellular redox state, and together with glutathione dismutase, Trx1 has been described as an important antioxidant agent leading to the protection of neuronal cells as DAn, by regulating the detrimental effects of oxidative stress and ROS formation [28]. Still, in vitro studies showed Trx1 as a regulator of apoptosis, demonstrating a modeling function on the activity of certain proteins such as the apoptosis signal-regulating kinase-1 (ASK-1) [29], thereby protecting DAn from dopamine-induced cell death (when exposed to 6-OHDA) due to its capability to reduce the neurotoxic dopamine metabolites, such as 6-OHDA-quinone [29]. Additionally, in an MPTP PD model, Trx1 was found to be an inducer of DAn cell survival, through the suppression of the endoplasmatic reticulum (ER) stress [30]. Nevertheless, although elucidation of the specific function of Trx1 on PD is required, human postmortem PD brains demonstrated that the levels of Trx1 were found to be significantly decreased, indicating that Trx1 secretion and function could be helpful for the study of new pathways and targets for PD repair [31]. Semaphorins, as SEMA7A, are secreted and transmembrane molecules that bind to plexin/neuropilin or integrin receptors, being involved in paracrine axonal guidance and development of functional neuronal networking [32,33]. Pacelli and colleagues [34] have described SEMA7A as a modulator of ROS-mediated neurodegeneration, by demonstrating that SEMA7A can reduce DAn axonal arborization and vulnerability through its capability to the decrease mitochondrial oxygen demanding and ROS production. Concerning UHCL1, ubiquitin plays a crucial role in the maintenance of the ubiquitin-proteasome system (UPS), which has been suggested as being involved in the initiation and progression of PD [35,36]. In fact, in PD brain tissue, UCHL1 is present in Lewy bodies, displaying an import role in maintaining UPS pool and DAn cell survival [36]. Nevertheless, although UCHL1 is involved in a genetic form of PD [37], its functions in (normal physiological conditions) living cells and tissues are still poorly understood, with several studies assuming UCHL-1 dysfunction associated to ubiquitinated proteins accumulation, as alpha-synuclein, the major cause of DAn cell degeneration [38,39]. Additionally, positive roles of UCHL1 in many other biological processes have also been claimed such as cell signaling, cell cycle, DNA repair, and other ubiquitination-dependent biological processes, which could represent additional routes for the establishment of therapeutic targets for the treatment of PD [38].

PEDF was previously described by our group as an important PD neurotrophic and neuroprotective molecule [16]. Such properties were tested and demonstrated by Falk and colleagues [40] who, by comparing PEDF with other PD therapeutic molecules (as GDNF), showed that PEDF has advantages in the ease of delivery and amelioration of functional outcomes. Similar results were then shown by Yasuda and colleagues [41] that demonstrated that PEDF levels increase in response to acute insults on the dopaminergic system, by correlating this response to the capacity of PEDF to interact and stimulate the activation of the NF-kB signaling cascade, which is involved in the modulation and expression of critical factors to DAn cell survival, namely BDNF and GDNF [40]. Regarding BDNF, studies have been described as a credible protective molecule in the degenerative process of PD, being an important molecule for the development, maturation, repair, and plasticity of DAn [42,43]. It has been demonstrated that the genetic inhibition of BDNF expression was correlated with a loss of nigral DAn [43], thereby indicating its importance in DAn cell survival/viability and neurite outgrowth as well [44]. Such pieces of evidence reinforce the importance of BDNF in PD initiation and progression, as it was defended that a decrease of BDNF expression in the SN might be one of the earlier steps at the onset of PD, by increasing DAn cells sensitization [45]. Regarding SDF-1, this factor is being positively correlated with beneficial effects on PD, being described as a promoter of DAn migration and neuritogenesis, which may constitute an interesting tool in PD regenerative medicine to improve (re)innervation of the nigrostriatal pathway [46]. Indeed, several studies have already highlighted that in addition to DAn migration, SDF-1 has an active role in the regulation of axonal pathfinding and elongation, thereby being considered a modulator of the dopaminergic system and functionality [46,47]. Looking to CypA, although the therapeutic effect of this protein under the PD context remains to be explored, few studies have indicated a potential role of it in the modulation of the ASK1 signaling pathway in oxidative stress-induced apoptosis through the inhibition of JNK and p38 activities [48,49]. Regarding Cys C, this protein has been largely explored as a promising therapeutic molecule under the context of Alzheimer’s disease, acting as a multitasking player in the induction of cellular autophagy, or via the inhibition of amyloid-β (Aβ) aggregation, leading to brain damage prevention [50,51]. Under the context of PD, only a few studies have already addressed the potential impact of Cys C [52,53]. For instance, Jing and colleagues [52] have recently demonstrated in A53T SNCA (alpha-synuclein overexpression model) transgenic mice that Cys C is involved in the neuroprotection of DAn, through a mechanism involving the upregulation of VEGF and NURR1 and the downregulation of Ser129-phosphorylated SNCA. Still, these authors also found that this neuroprotective effect could also be explained by the active role of Cys C in autophagy, as demonstrated by the upregulated levels of LCB3 in Cys C-treated animals. Similar outcomes were previously presented by Xu and colleagues [53], who found Cys C as a survival promoter of DAn after 6-OHDA insult both in vitro and in vivo, suggesting that Cys C may play an important role in brain self-protection following injury. Concerning VEGF, and it was already mentioned, this factor plays an important role in the neurorescuing of DAn, by interacting with different players in the potentiation of the dopaminergic system [52,54]. Similarly, it is DJ-1 that has been described as a major player and target for PD modeling and repair [2,55,56]. Notably, studies have demonstrated that DJ-1 deficiency sensitizes microglial cells to release pro-inflammatory cytokines (e.g. IFN-γ and I- TAC), causing inflammatory damage to DAn, thereby indicating that DJ-1 could be a potential key molecule to tackle PD [57]. Besides, DJ-1 was also found to be a modulator of mitochondrial function [58]. Chen and colleagues [58], have recently shown that DJ-1 can directly bind to the F1FO ATP synthase β subunit, decreasing mitochondrial uncoupling and enhancing ATP production efficiency, correlating such outcomes with an enhancement of dopaminergic cell metabolism and growth [58]. Interestingly, in addition to these anti-inflammatory and mitochondrial functional effects, DJ-1 has also been described as a PD antioxidant agent, by regulating specific transcription factors involved in the increase of the Trx and GSH redox systems, which in turn can also modulate DJ-1 functionality [56,59]. GDNF, in contrast, is a well-established neurotrophic agent for DAn survival, viability, and functionality both in vitro and in vivo, by maintaining its morphology and neurochemical phenotype [60]. From the application point of view, and given, for instance, prior 6-OHDA administration, GDNF demonstrated to be effective in the protection of striatal DAn [61]. In addition to neuroprotection, GDNF has also been found as a modulator of apoptosis, by presenting an active role in upregulating anti-apoptotic proteins such as Bcl2 and Bcl-X through PI3K signaling pathway [42,60,62]. Still, GDNF has also been described as an inducer of antioxidant activities, as it was found to be able to positively increase the activity superoxide dismutase, catalase, and glutathione peroxidase, allowing the detoxification of ROS-induced degeneration [63]. Even under clinical trials, GDNF has presented promising results, although more studies should be explored to further address its full potentiality [64]. Following GDNF effects, it was interesting to find a connection with CDH2, which according with Zuo and colleagues [65] appears to be a mediator and an effector of GDNF actions in the activation of the PI3k/Akt signaling pathway, which has been correlated with protective effects on DAn. Indeed, the same authors also demonstrated that when they knocked down CHD2, the protective effects of GDNF was diminished, hypothesizing that CDH2 is linked to the biological effects of GDNF in DAn [65]. In line with this, Sakane and Miyamoto [66] demonstrated that CDH2 has also important functions in DAn differentiation, by demonstrating that it regulates Wnt-b-catenin signaling and controls the proliferation and differentiation processes of DAn progenitors in the ventral midbrain region. Regarding IL-6, studies have shown that its presence in MSCs secretome plays important roles in scavenging ROS, as superoxide radicals, by increasing the antioxidant enzyme activity, through STAT pathways, and leading to DAn protection [67]. In addition to this antioxidant activity, IL-6 has also been linked to DAn neuroprotection [68]. Indeed, studies have demonstrated that knocking down IL-6 increases the vulnerability of DAn to MPTP, by speculating that IL-6 is capable of protecting DAn from the MPP^+^-induced toxicity [69,70]. Considering Hsp27, Lee and colleagues [71] have described this molecule as a protective agent of DAn, due to its capability to attenuate alpha-synuclein-induced toxicity. In line with this, Cox and colleagues [72] demonstrated that Hsp27 can directly bind to alpha-synuclein fibrils, thereby preventing or disrupting the onset and progression of its aggregation and consequent debilitating effects on DAn. Concerning PRDX1, it has been presented as a potent antioxidant agent, promoting DAn cell survival and protection from toxic insults as 6-OHDA, respectively [73]. The modulation of p38-MAPK and caspase-3 signaling pathways is being defined as the molecular mechanism by which PRDX1 exerts a protective role in experimental models of PD, further supporting it as an important regulator of the (DAn) cell death pathway [73,74]. Finally, GDN is being described as an interesting molecule playing crucial roles in the enhancement of neurite outgrowth and neuroprotection through the prevention of oxidative stress [75,76]. Nevertheless, although Clusterin, CypB, Gal-1, UBE3A, and MMP-2 were not found to be directly interconnected, its presence on MSCs secretome has also been correlated with positive and promising effects on PD modeling and repair, namely through the modulation of oxidative stress and neuroinflammation, mitochondrial dysfunction prevention, and alpha-synuclein degradation, thereby exerting neuroprotective properties in PD-related environments [77,78,79,80,81].

Considering levodopa effects, such pieces of evidence (as seen in hBM-MSCs secretome application) were not, in contrast, observed in the treated animals. In fact, the pharmacological application of levodopa (even being the standard treatment of PD) remains still controversial, due to an inconsistency of results regarding its truly mechanistic effects [82]. Of note, while in vitro studies have demonstrated both toxic (through dopamine metabolism and autoxidation, giving rise to quinones and hydrogen peroxide) and protective effects (at certain concentrations) of levodopa on DAn, in vivo and even clinical studies have not provided yet any convincing data [82]. Inevitably, levodopa dose-regiment is, indeed, a critical issue in treating PD [83,84]. Colamartino and colleagues [82] have hypothesized that one of the most difficult aspects of experimental analysis regarding levodopa treatment is related to the individual response of each subject, both regarding the effectiveness of therapy and its side effects. Additionally, it has also been defended that this discrepancy of results could, most likely, be due to the intrinsic nature of the molecule of levodopa, which has both pro-oxidant and antioxidant properties that depend on the concentrations used [85]. Preclinical studies have demonstrated that while acute treatments with levodopa (as used in the present study) might not be able to restore motor activity, chronic administration appears to be more effective in reducing parkinsonian motor deficits and in restoring striatal synaptic functionality [86]. However, this therapeutic efficacy of levodopa is just remarkable in the first years of treatment if a conceivable presence (in sufficient number) of spared DAn exists, which allows the conversion of levodopa and mediate the physiological release of dopamine [87]. Notwithstanding, as PD progresses, the responsiveness to levodopa declines over time, thereby requiring an increase in dosage, which in the majority of the cases is coincident with a more rapid reduction in levodopa efficacy at the end of dose, known as the ‘off-phase’ [88], which could explain the poor performance of our levodopa-treated animals (Figure 3). Therefore, despite its longevity, it becomes clear that understanding the mechanism underlying both therapeutic and negatives effects of levodopa is critical [89], as it could open new questions and hypotheses about alternative pathways for better understanding the etiology and pathophysiology of PD [90]. 

In conclusion, the present findings support hBM-MSCs secretome administration as a potential therapeutic tool in treating PD. Although we suggest candidate molecules and possible mechanisms of hBM-MSCs secretome-mediated effects, further detailed studies are needed to carefully and clearly define which players may be responsible for its therapeutic actions. Still, although no significant differences were observed for levodopa, additional work is necessary to improve the applicability of this approach. For instance, the route of administration of hBM-MSCs secretome should be revised. Of note, instead of intracranial injections (which is an invasive protocol from the clinical point of view), intravenous injections should be considered, which could represent a more reliable way to make a ‘direct’ comparison with levodopa administration. Still, as to date, no regenerative/neuroprotective strategy was already approved as a PD therapy [91], a fact that can be partially attributed to the ‘one-disease-one-target’ view that has been followed [92], the development of combinatorial strategies may overcome the limitations of single drug approaches, particularly by combining the latter (as levodopa) with stem cells secretome. By doing so, it will be reasonable to presume that potential treatments than can, per se, or in combination modulate or slow PD progression will be the result of a better understanding of PD pathophysiology.

## 5. Conclusions

Re-establishing DA level equilibrium, nigrostriatal function and integrity and, with it, regaining the loss of sensory-motor functionality is one of the main challenges of PD regenerative medicine. By comparing hBM-MSC secretome and levodopa applications independently, although no differences were observed between them, we observed a significant amelioration in the animal behavior and (DAn) histological densities of secretome-treated animals when compared to those untreated (6-OHDA). Although the underlying mode-of-action of hBM-MSCs secretome remains unexplored, we hypothesize it as a multitargeted modulator of different neural mechanisms triggered by presence of multifactorial proteic composition such as Trx1, SEMA7A, UCHL1, PEDF, BDNF, Clusterin, SDF-1, CypA, CypB, Cys C, VEGF, DJ-1, Gal-1, GDNF, CDH2, IL-6, HSP27, PRDX1, UBE3A, MMP-2, and GDN, respectively. Therefore, our findings suggest that the stimulation of PD histological and behavioral deficits promoted by the secretome appears to not be dependent on the presence of one secreted factor, but from an integrated interaction of several, allowing the gaining of new insights concerning the potential interplay of these soluble factors and their neuroprotective, antioxidant, anti-apoptotic, and DAn homeostatic effects, which may lead to a rational design of new therapeutic or adjuvant strategies for the functional recovery of PD.
